# Breast cancer care in Uganda: A multicenter study on the frequency of breast cancer surgery in relation to the incidence of breast cancer

**DOI:** 10.1371/journal.pone.0219601

**Published:** 2019-07-11

**Authors:** Tove Ekdahl Hjelm, Alphonsus Matovu, Noleb Mugisha, Jenny Löfgren

**Affiliations:** 1 Department of Oncology, Stockholm South General Hospital, Stockholm, Sweden; 2 Mubende Regional Referral Hospital, Mubende, Uganda; 3 Department of Oncology, Uganda Cancer Institute, Kampala, Uganda; 4 Department of Molecular Medicine and Surgery, Karolinska Institute, Stockholm, Sweden; Duke University, UNITED STATES

## Abstract

**Background:**

Breast cancer is the most common cancer in women worldwide. Considerable funding and efforts are invested in breast cancer research and healthcare, but only a fraction of this reaches women and healthcare systems in low income countries. Surgical treatment is an essential part of breast cancer care, but access to surgery is in general very limited in low income countries such as Uganda. In this study, the previously unknown nationwide rate of breast cancer surgery was investigated.

**Methods and findings:**

This was a multicenter, retrospective study, investigating breast cancer surgery in the public healthcare system in Uganda. Data were collected from operating theater registries at primary, secondary and tertiary level healthcare centres throught the country, including 14 general hospitals, the 14 regional referral hospitals and the national referral hospital. Patients who underwent major surgery for breast cancer at these hospitals during 2013 and 2014 were included. The number of breast cancer procedures performed, geographical variation, level of healthcare staff performing surgery and patient characteristics were investigated. After correction for missing data, a total of 137 breast cancer procedures were performed each year within the public healthcare system, corresponding to 5.7% of the breast cancer incidence in the country at that time. Most procedures (n = 161, 59.0%) were performed at the national referral hospital by qualified surgeons. Many of the patients were young; 30.1% being less than 40 years old. The proportion of male breast cancers in the study was large (6.2%).

**Conclusions:**

The rate of breast cancer surgery in Uganda is minimal and in several parts of the country breast cancer surgery is not performed at all. More resources must be directed towards breast cancer in low income countries such as Uganda. The fact that the patients were young calls for further research, prevention and treatment specifically targeting young women in the study setting.

## Introduction/Background

Non-communicable diseases (NCDs) including malignancies, are becoming a major health concern in low- and middle-income countries (LMICs) [1,2]. In 2018, 18.1 million new cancers are expected to be diagnosed and 9.6 million cancer related deaths will occur worldwide [3], of these cancer-related deaths 65–70% occur in a LMIC [4,5]. In the third edition of Disease Control Priorities published by the World Bank, support for research on cancer in LMICs, *i*.*e*. to strengthen the capacity to deal with the cancer burden, is pointed out as being a worthwhile investment for high income countries (HICs) [6]. At least 80% of all cancer patients require surgery for their treatment. The Lancet Oncology Commission recommends that cancer disease that carries a large disease burden and where surgery has a vital role should be given priority [7]. Breast cancer fits this description.

Breast cancer is the commonest form of cancer in women worldwide and its associated disease burden increased by over 35% between 1990 and 2010 [8]. Breast cancer patients in LMICs are younger than those in HICs and often present with advanced disease [9,10]. This contributes to high mortality rates, and the documented overall survival rate for breast cancer patients does not exceed 60% in any LMIC in Africa [11]. The opportunities for research and improvement of surgical and oncological services in LMIC are extensive and much needed.

Uganda is a low-income country in sub-Saharan Africa (SSA) with a total population of 36.3 million people in 2012 [12]. The population is young with a life expectancy at birth of 64 years for women [13]. In the GLOBOCAN 2012 and 2018 projects, available data were used to estimate incidence, prevalence and mortality of cancer worldwide. Breast cancer is the second most common cancer in Uganda after cervical cancer, according to both GLOBOCAN 2012 and 2018 [3, 14]. The 5-year survival rate for patients with breast cancer has been shown in previous Ugandan studies to be 46–56%, compared to 87.7% in Sweden [11,15–17]. Surgery is a basic part of breast cancer healthcare programs and is necessary in any curative attempt. A small fraction of surgical procedures in the world are conducted in LMICs [18]. A study from a regional referral hospital in Uganda showed that 11.3% of the patients admitted for surgery had a cancer diagnosis, and 1.2% of all patients were admitted because of breast cancer [19]. The volume of breast cancer surgery being performed in Uganda on a national scale is not known. A major problem in Uganda, as in many LMICs, is the lack of primary data. Nationwide cancer or surgical registries do not exist [19]. In this study, a national survey of breast cancer surgery at primary, secondary and tertiary level hospitals in Uganda was performed.

## Materials and method

### Study design

This was a multicenter, retrospective, facility-based study.

### Data collection

**Fig 1:**
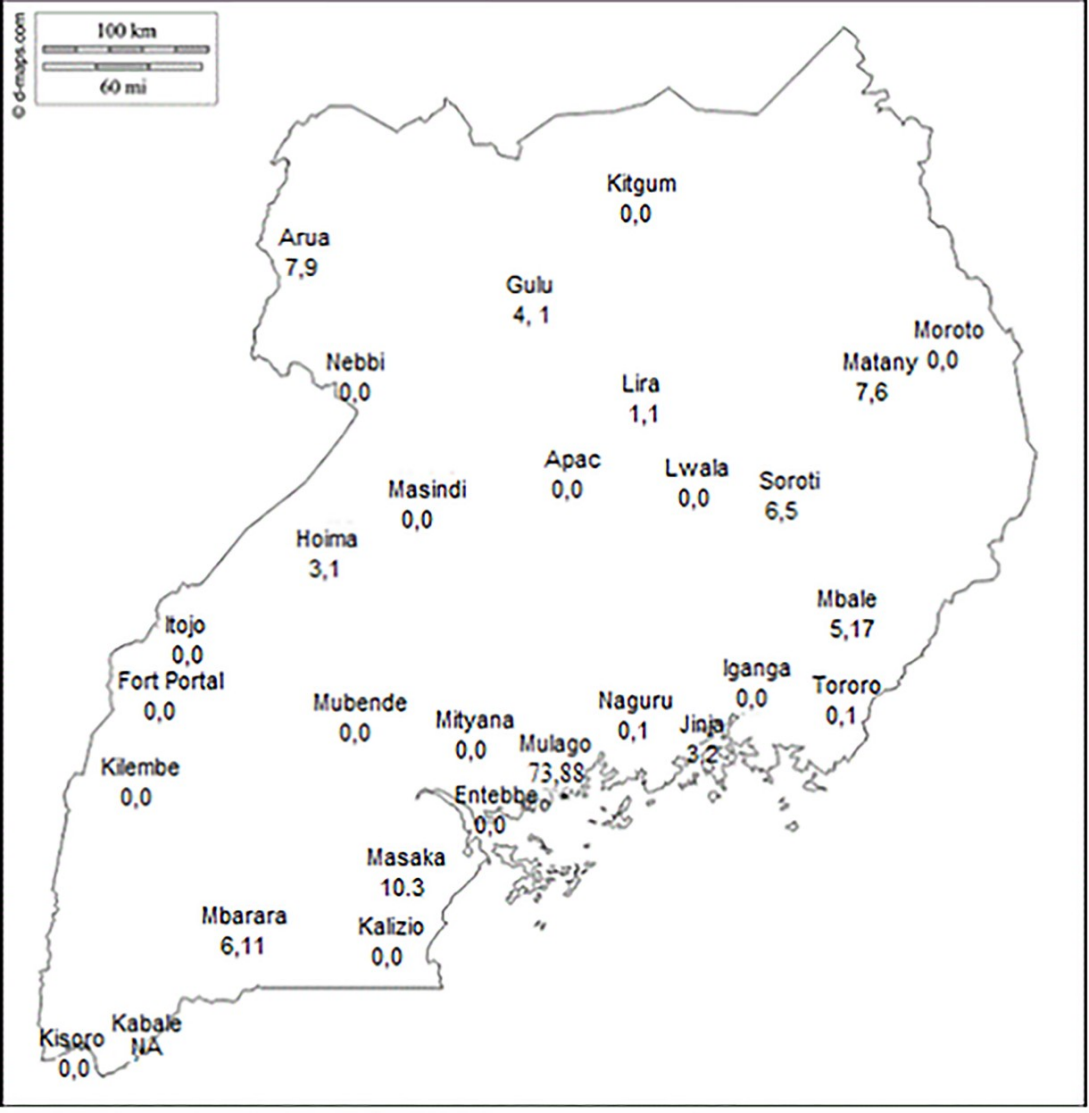
Map of geographical distribution of hospitals participating in the study and volume of breast surgery performed 2013–2014.

There is one national referral hospital (Kampala), 14 regional referral hospitals (RRH) and 139 general hospitals (GH) in Uganda. Data collection was carried out at the national referral hospital (NRH), all 14 regional referral hospitals (RRH) and 14 general (district) hospitals (GH) in Uganda; one GH for each healthcare region (Fig 1). All hospitals, excluding two missionary hospitals (GH), worked within the public healthcare system. Data were retrieved from operating theater records for the time period January 1 2013 to December 31 2014 and included date of surgery, age and sex of patient, diagnosis, surgical procedure and surgeon performing the operation. Breast cancer was defined as the diagnosis assigned in the theater registry by the surgeon. Major surgical procedures included tumor/breast excisions, debulking/debridement, quadrantectomies, lumpectomies, simple-, radical- and palliative/toilet mastectomies. Minor procedures such as breast biopsy were excluded. Data on breast cancer surgery were extracted and entered onto Excel spread sheets.

### Data analysis

Descriptive statistical methods were used to calculate volumes and describe the characteristics of the patients and the procedures. Missing numbers for breast surgery in the RRH and the NRH were handled by multiple imputations (20 imputations in total). The imputation numbers were created using non-missing numbers. Imputations were averaged, and numbers rounded off, to give a single estimate of the number of breast procedures for the month in question. The maximum number of procedures expected to be performed each month was estimated in the imputation to be 15 cases.

### Ethical implications

The study was approved by the IRB at the School of Public Health at Makerere University (HDREC 076) and by the Uganda National Council of Science and Technology (HS 1892). The Medical Superintendent of each hospital approved data collection from the theater registries. In this study, operation theatre log books were used as the source of information. This is a log book where information about the patient, the procedure and the staff (surgeon, assistant, anaesthetist) is included. Photos were made of all records available and before making these photos the names of the patients were covered. No individual patient records were used. The study was retrospective and therefore did not involve direct contact with patients and there was no direct risk to the patients. Anonymity was ensured by not recording the names of the patients.

## Results

### Volumes and rates

Information on breast cancer surgery was retrieved from all hospitals in the survey but one, where data on general surgery were not available in the hospital theater records. This hospital, Kabale RRH, was excluded from data analysis. Data for the whole of 2014 were missing at the NRH. In total, data for 21 months were missing from the RRHs and 26 months were missing from the GHs. Data for a total of 291 months were available at the RRHs and for 310 months at the GHs (S1 and S2 Tables). A total of 182 surgical procedures for breast cancer were recorded. After correction for missing data using imputation, the number was 273 (Table 1 and S3 Table) giving an annual breast cancer surgery rate of 137.

**Table 1:** Breast cancer surgery in General, Regional Referral and the National Referral Hospitals in 2013 and 2014.

	General Hospital		Regional Referral Hospital		National Referral Hospital	
Year	2013	2014	2013	2014	2013	2014
N (%)	7 (5.6)	7 (4.7)	45 (36∙0)	53 (35.8)	73 (58.4)	88 (59.5)
Range	0–7		0–17		73–88	
Mean (SD)	0.5 (1.7)		3.8 (4.2)		80.5 (10.6)	
Median (IQR)	0 (0)		2.5 (5.3)		80.5 (-)	

Most of the procedures (n = 161, 59.0%) were performed at the NRH followed by the RRHs (n = 98, 35,9%). Very little breast surgery was undertaken at the GH level. Large areas of the country had no breast surgery at all (Fig 1). Rates varied between 0 and 0.55 per 100,000 population per year. The proportion of surgery, catchment population, breast cancer incidence for each of the RRHs and the NRH are presented in Table 2.

**Table 2:** Proportions of procedures, catchment population, breast cancer incidence and met need at Regional Referral Hospitals and the National Referral Hospital.

Hospital	Operations		Catchment population, million (reference)	Breast cancer incidence (n)	Estimated need (n)	Met need (%)
	*Mean count per year*	*Proportion of total (%)*				
**Arua RRH**	8	5.7	3.5 (18)	235	188	4.3
**Fort Portal RRH**	0	0	2 (3)	134	107	0
**Gulu RRH**	2.5	1.8	2 (3)	134	107	2.3
**Hoima RRH**	2	1.5	3 (18)	201	161	1.2
**Jinja RRH**	2.5	1.8	2 (3)	134	107	2.3
**Kabale RRH**	NA	NA	2 (3)	134	107	NA
**Lira RRH**	1	0.7	2 (3)	134	107	0.9
**Masaka RRH**	6.5	4.8	2 (3)	134	107	6.1
**Mbale RRH**	11	8.1	2 (3)	134	107	10.3
**Mbarara RRH**	8.5	6.2	4 (18)	268	214	4.0
**Moroto RRH**	0	0	1.3 (18)	87	70	0
**Mubende RRH**	0	0	2 (3)	134	107	0
**Naguru RRH**	0.5	3.7	3 (4)	201	161	0.3
**Soroti RRH**	5.5	4.0	2 (3)	134	107	5.1
**Mulago NRH**	80.5	58.9	36.3 million (13)	2420	1936	4.2

Detailed information about catchment population were entered for the RRH where information could be found, for the other RRH the catchment population was entered as two million, which is the target population for RRH (27). The incidence for breast cancer used was 2420 per 36.3 million, (breast cancer incidence according to GLOBOCAN 2012), which equals 6.7 per 100 000 population [14].

The majority of patients were female (n = 150, 93.8%), 10 patients (6.2%) were male and in 22 cases gender information was missing. The patients were young; 14 patients (8.6%) were aged between 20 and 29 years, and 35 patients (21.5%) between 30 and 39 years (Table 3). The mean age was 48 years (SD 5.7). For 19 patients age was not specified.

**Table 3:** Age distribution of breast cancer patients 2013 and 2014.

Age	Number (%)
0–19	0 (0)
20–29	14 (8.6)
30–39	35 (21.5)
40–49	38 (23.3)
50–59	30 (18.4)
60–69	31(19.0)
>70	15(9.2)
Age n/a	19 (11.7)

The incidence of breast cancer in Uganda for both sexes was 2420 for the year 2012, according to the GLOBOCAN 2012 [14]. Assuming this figure to be true for the years 2013 and 2014, and the annual rate of 137 breast cancer procedures found in our survey, we estimate that only 5.7% of expected new breast cancer cases in Uganda receive surgical attention.

### Met and unmet need for surgery

According to the Lancet Commission of Global Oncology, the estimated proportion of breast cancer patients who need surgical intervention is 80% [7] implying that the annual need for breast cancer surgery in Uganda is 80% of the incidence *i*.*e*. 1936 cases. Since only 137 cases were operated each year during the study period, the estimated met need for breast cancer surgery performed within the public healthcare sector was 7.1% for the years 2013 and 2014 and hence the unmet need for breast cancer surgery was 92.9%. The met need for breast cancer surgery in the RRHs varied between 0% and 1.3% (Table 2).

### Human resources

The majority of operations were performed by a qualified surgeon (n = 120, 69.4%), but were also performed by residents in surgery, medical officers and intern doctors (Table 4). A Medical Officer, is a medical doctor who has a licence to practice (has completed internship after medical school) but who does not have any specialist training. In nine cases the person performing surgery was not defined.

**Table 4:** Category of medical staff performing breast cancer surgery.

Level of hospital	Surgeon (n)	Resident in surgery (n)	Medical Officer (n)	Intern Doctor (n)
National Referral Hospital, Mulago	56	12	0	0
Regional Referral Hospitals	59	3	29	4
General Hospitals	5	2	3	0
**Total (%)**	**120 (69.4)**	**17 (9.8)**	**32 (18.5)**	**4 (2.3)**

Information about medical staff performing surgery was missing in nine cases.

## Discussion

This survey revealed an annual rate of 137 breast cancer procedures performed within the public healthcare system of Uganda, corresponding to 5.7% of the breast cancer incidence in the country. Breast cancer surgery is mainly performed at the NRH in the capital city of Kampala where a minority of Uganda’s population resides. Women undergoing breast cancer surgery were young with a mean age of only 48 years. Almost a third of the patients were below the age of 40. The proportion of male breast cancers was high, 6.2%, compared to less than 1% of breast cancer cases reported in previous epidemiologic studies on breast cancer [20]. Uganda was used as a typical low-income country in SSA, and the situation is presumably similar in other LMICs in Africa.

There are several reasons for the low frequency of breast cancer surgery in LMICs including: late stage at diagnosis; low breast cancer awareness in the society; long waiting lists for surgery; difficulties in navigating through the healthcare system; widespread use of alternative medicine; economic factors; and the fear or stigma of mastectomy all of which have been identified in previous studies [21]. The fact that many patients present at an advanced stage limits breast-conserving surgery, meaning that most patients are in need of radical mastectomy if a curative attempts are still feasible [22]. Furthermore, the limited access to radiation therapy contributes to the low frequency of breast-conserving surgery. Other explanations for the low rate of breast cancer surgery are related to an inadequately financed healthcare system and the lack of trained surgeons in LMICs where healthcare resources and skilled personnel are often restricted to the main cities, thus limiting access to care in rural areas. The Lancet Commission on Global Surgery recommends 20 to 40 surgical providers (including specialist surgeons, obstetric physicians and anesthetists) per 100,000 inhabitants, evenly distributed between rural and urban areas [18]. In Uganda, around 100 surgeons serve the whole population of close to 40 million people, and 95% of these work in urban areas despite 80% of the population living in rural areas [19]. In South Africa it has been shown that for each 30 km additional distance between the treatment facility and the patient’s home, the risk of presenting with metastatic disease increased by 25% [23].

The finding in this survey that patients were young is consistent with those of previous studies from Uganda and other LMICs in SSA, which have shown women in these countries on average present with breast cancer 15 years earlier than women in Europe and North America [18]. The reasons behind this are not yet fully understood. Demographic differences including a younger population and shorter life expectancy compared to HICs could be part of the explanation, but other factors may also play a role. It has been established that breast cancer before the age of 40 is often linked to genetic factors such as mutations in the breast cancer (BRCA) genes 1 and 2 [24]. It is possible that gene mutation is an important risk factor for breast cancer disease in SSA but this has not been adequately investigated. Small studies from Nigeria and Sudan have shown an incidence of BRCA 1/2 gene mutation in early onset breast cancer (<40 years) averaging between 2.6 and 14.3% [25–26]. Further research in this field may shed more light on the cause of breast cancer in SSA. Close monitoring and possibly prophylactic medical and or surgical treatment of individuals with elevated risk for developing breast cancer could be an option in LMICs where nationwide screening will not be affordable for many years to come.

Early detection is a key factor in achieving lower mortality rates from breast cancer. Mortality rates approaching 60% in SSA are not reasonable when 5-year survival in HICs are close to or above 90%. This is the result of global inequality in cancer care. The World Health Organization has reported that LMICs account for only 10% of the total expenditure of cancer control and care, even though 50% of the world’s cancer patients live in these countries [9]. Given the considerable funding available for breast cancer research and care worldwide, making changes for the better in LMICs is not only desirable but also highly feasible.

Surgery is an important part of treatment for breast cancer, but parallel investments in other oncologic fields such as chemotherapy, radiation therapy and hormonal therapy, together with investments in palliative care and cancer research, is essential if we are to increase the quality of cancer care globally.

### Strengths and limitations

This study represents breast cancer surgery in the public healthcare system in Uganda. The study is a nationwide survey with data collected over a period of two years from public primary, secondary and tertiary level healthcare centers in all regions of the country. Two mission hospitals were included where no suitable public general hospital was present. It is possible that additional breast surgery may have been carried out in private hospitals, though this is unlikely as most people in Uganda lack the means to afford private healthcare. The present study thus represents a fair picture of breast cancer surgery volumes nationwide in Uganda.

The weaknesses of this study are mainly related to availability and level of detail of the data processed; a typical example being that lack of detail meant breast cancer diagnoses could not be verified. Breast cancer cases were defined as the assigned diagnosis entered by the surgeon in the theater registry. There was no possibility to verify the diagnosis histologically in this survey. Previous studies have estimated that 10–50% of cancer cases in Uganda never have pathology performed, meaning that up to 50% of breast cancer cases could be missed if we included only those with a histologically verified diagnosis [27]. As information from some time periods were missing in the theater registries (as specified in the results section), imputation was used to compensate for missing data. Unfortunately, there is generally lack of nationwide population-based cancer registries in SSA, and data reported from population- and hospital registries are often inconsistent and of poor quality. Missing data and lack of detailed data is common [28]. To establish nationwide cancer- and surgical registries in these countries would be very helpful for research purposes and for policy makers to guide resources for future health care investments.

The real need for breast cancer surgery in this study was estimated to be 80% of the incidence according to GLOBOCAN 2012. This is possibly an underestimation of the need for surgery since almost all newly diagnosed breast cancer patients should need surgery, if they were to be diagnosed in time. In this survey we estimated the unmet need for breast surgery in LMICs to be around 93%, though following the logic mentioned above, this figure might be even higher. There are to our knowledge no previous study investigating the met need for breast cancer surgery in any LMIC in SSA. Though, in a review article published in Journal of Global Oncology, the rate of surgical treatment for breast cancer varies widely between studies and countries in Africa, the majority stating surgical rates from 48–75% of diagnosed cases [29].

## Conclusions

Breast cancer in LMICs is increasing rapidly, between 1990 and 2013 the incidence per 100,000 inhabitants in developing countries increased by 46% [30]. This fact together with the already large unmet need for breast cancer surgery found in this study means there really is an urgent need for investment in breast healthcare in these countries, to increase access to surgical services and improve outcome for breast cancer patients.

## Supporting information

S1 Table Volume of breast cancer surgery for each hospital and month 2013. Data before imputation was performed. The hospital Kabale was excluded from data analysis due to total missing data for 2013 and 2014.
(XLTX)

S2 Table Volume of breast cancer surgery for each hospital and month 2014. Data before imputation was performed. The hospital Kabale was excluded from data analysis due to total missing data for 2013 and 2014.
(XLT)

S3 Table Imputated data. *Imputated figure.
(XLTX)
